# A mild impairment in reversal learning in a bowl‐digging substrate deterministic task but not other cognitive tests in the Dlg2+/− rat model of genetic risk for psychiatric disorder

**DOI:** 10.1111/gbb.12865

**Published:** 2023-09-13

**Authors:** Simonas Griesius, Sophie Waldron, Katie A. Kamenish, Nick Cherbanich, Lawrence S. Wilkinson, Kerrie L. Thomas, Jeremy Hall, Jack R. Mellor, Dominic M. Dwyer, Emma S. J. Robinson

**Affiliations:** ^1^ Centre for Synaptic Plasticity, School of Physiology, Pharmacology and Neuroscience, University of Bristol, University Walk Bristol UK; ^2^ Neuroscience and Mental Health Research Institute, Psychology Cardiff UK; ^3^ Department of Psychology Cardiff UK; ^4^ MRC Centre for Neuropsychiatric Genetics and Genomics, Schools of Medicine and Genetics and Genomics, Schools of Medicine and Psychology Cardiff UK; ^5^ Department of Medicine and Psychology Cardiff UK

**Keywords:** autism spectrum disorder, Chapsyn‐110, cognition, MAGUK, PSD‐93, reversal learning, schizophrenia

## Abstract

Variations in the Dlg2 gene have been linked to increased risk for psychiatric disorders, including schizophrenia, autism spectrum disorders, intellectual disability, bipolar disorder, attention deficit hyperactivity disorder, and pubertal disorders. Recent studies have reported disrupted brain circuit function and behaviour in models of Dlg2 knockout and haploinsufficiency. Specifically, deficits in hippocampal synaptic plasticity were found in heterozygous Dlg2+/− rats suggesting impacts on hippocampal dependent learning and cognitive flexibility. Here, we tested these predicted effects with a behavioural characterisation of the heterozygous Dlg2+/− rat model. Dlg2+/− rats exhibited a specific, mild impairment in reversal learning in a substrate deterministic bowl‐digging reversal learning task. The performance of Dlg2+/− rats in other bowl digging task, visual discrimination and reversal, novel object preference, novel location preference, spontaneous alternation, modified progressive ratio, and novelty‐suppressed feeding test were not impaired. These findings suggest that despite altered brain circuit function, behaviour across different domains is relatively intact in Dlg2+/− rats, with the deficits being specific to only one test of cognitive flexibility. The specific behavioural phenotype seen in this Dlg2+/− model may capture features of the clinical presentation associated with variation in the Dlg2 gene.

## Graphical abstract

Variations in the Dlg2 gene have been linked to increased risk for psychiatric disorders. Here, we tested these predicted effects with a behavioural characterisation of the heterozygous Dlg2+/‐ rat model. Dlg2+/‐ rats exhibited a mild impairment in reversal learning but only in a substrate deterministic bowl‐digging reversal learning task.

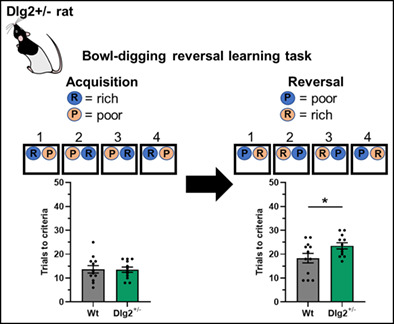

## 1 INTRODUCTION

Genetic variation in the Dlg2 gene, which encodes the membrane associated guanylate kinase (MAGUK) synaptic scaffolding protein PSD93, also known as Dlg2 or chapsyn‐110, has been associated with an array of psychiatric disorders^1^ including schizophrenia,^2^ intellectual disability,^3^ bipolar disorder,^4^ autism spectrum disorder,^5^ attention deficit hyperactivity disorder,^6^ and pubertal disorders.^7^, ^8^ The PSD93 protein is expressed widely throughout the brain^9^, ^10^, ^11^and has been demonstrated to interact with a variety of other proteins in the postsynaptic density, including NMDAR receptor subunits,^12^, ^13^, ^14^, ^15^, ^16^ AMPAR auxiliary subunit stargazin,^17^ potassium channels,^18^, ^19^, ^20^, ^21^ and other cytoskeletal components.^12^, ^22^, ^23^, ^24^, ^25^, ^26^ Work on homozygous knockout rodents models of Dlg2−/− has found evidence of deficits in glutamatergic signalling,^10^, ^14^, ^27^, ^28^, ^29^, ^30^, ^31^ plasticity,^28^ and dendritic^32^ and spine^31^ morphology in the hippocampus, dorsal striatum, spinal dorsal horn, and cingular and visual cortices. We have recently demonstrated in the heterozygous rat Dlg2+/− model hippocampal CA1 pyramidal neurons enhanced NMDA receptor currents, reduced input resistance, reduced dendritic arborisation, impaired dendritic integration, and associative plasticity.^33^

At the behavioural level, deficits in sociability^10^, ^34^ and hypoactivity in response to novelty,^10^, ^34^ deficits in cognitive flexibility and attention,^35^ and impaired motor learning and coordination^34^ have been found in homozygous Dlg2−/− models. In heterozygous Dlg2+/− models, which may arguably more closely resemble the haploinsufficiency found clinically, these phenotypes have generally been milder, with task performance indistinguishable from wild‐types in some cases.^10^ Dlg2+/− heterozygous mice show impairments in motor learning and habituation to novel contexts.^36^ We have recently reported that Dlg2+/− heterozygous rats exhibit an enhanced locomotor response to phencyclidine but are indistinguishable from wild‐types in tests of anxiety, hedonic reactions, social behaviour, and sensorimotor gating.^37^

Due to the widespread expression of PSD93, various behaviours could be affected in models of PSD93 deficiency. In these studies, we extend the characterisation of the Dlg2+/− heterozygous rat model with a focus on cognitive function and behavioural tests associated with different forms of learning and memory and cognitive flexibility. We chose to complement our previous behavioural characterisation of the Dlg2+/− rat model with tasks focusing on learning and memory, and specifically reversal learning tasks to investigate cognitive flexibility.^38^, ^39^, ^40^, ^41^, ^42^, ^43^, ^44^, ^45^ Impairments in cognitive flexibility have been demonstrated in patient populations, including those suffering from schizophrenia,^41^, ^44^, ^45^ autism spectrum disorder,^38^, ^39^ and attention deficit hyperactivity disorder,^40^, ^43^ for which genetic variations in the Dlg2 gene are a risk factor for. The hippocampus has been suggested to contribute to reversal learning in humans.^46^, ^47^ We also recently reported changes in the physiology of circuits in the Dlg2+/− model which are specific to the hippocampus.^33^ We chose to use a mixture of tasks involving either prolonged training in the touchscreen apparatus, more naturalistic foraging‐based learning tasks using bowl‐digging or exploration‐based tasks. We report a specific deficit in reversal learning in a bowl‐digging reversal learning task whilst performance in a touchscreen reversal learning task, visual discrimination, novel object and novel location preference, spontaneous alternation, modified progressive ratio, and novelty suppressed feeding are all unaffected in the Dlg2+/− rats. These finding suggest that this Dlg2+/− heterozygous rat model exhibits a specific but subtle behavioural impairment associated with cognitive flexibility.

## 2 MATERIALS AND METHODS

### 2.1 Animals and husbandry

Heterozygous Dlg2+/− rats (Long Evans Hooded background) were generated by Horizon Discovery (Pennsylvania, USA) using CRISPR/Cas9 gene editing technology. A 7 bp deletion (782933–782,939 in the genomic sequence) in exon 5 caused a frame shift and an early stop codon in exon 6. Heterozygous founders were sent to Charles River (Margate, UK) and bred to produce experimental colonies by breeding male heterozygous rats with female wild‐types, producing a 50/50 distribution of heterozygous and wild‐type offspring. More detail is available in Supplement 1 of Griesius et al. 2022.^33^

Behavioural tests were carried out on a total of 4 cohorts of wild type and Dlg2+/− female and male rats at the University of Bristol and Cardiff University (Supplementary Figure S1). Cohort 1 (Bristol) consisted of 12 Dlg2+/− and 12 wild‐type male rats. Cohort 2 (Cardiff) consisted of 20 Dlg+/− and 28 wild‐type male rats. Cohort 3 (Cardiff) consisted of 21 Dlg+/− and 24 wild‐type female and male rats. Cohort 4 (Cardiff) consisted of 24 Dlg+/− and 40 wild‐type female and male rats. The results from three tasks were not included in the analysis following a failure to establish learning in the wildtype animals. This was the object in place task, temporal order task and water maze learning and reversal task. In the object in place and temporal order tasks, wild‐type animals failed to achieve discrimination, mean discrimination ratio was less than 0.1 and was not significantly different from chance (one‐sample *t*‐test against a theoretical mean of 0). In the 10‐day reference and memory in water maze, wild‐type animals failed to show memory of the platform location on the day 5 probe test by searching for the platform in the quadrant it had been at during training. These cohorts carried out batteries of tasks in different domains, designed to extensively characterise the Dlg2+/− rat model. The results of the tasks not reported in this article can be found in Waldron et al. 2022.^37^ Approximately equal numbers of male and female rats were used in cohorts 3–4. Cohort 1 and 2 consisted of male rats only. Cohort number does not indicate the order in which the cohorts were generated or experiments carried out. Groups sizes across cohorts were determined using published studies assessing between‐subject effects, pilot data, task type, as well as welfare and practical considerations. Sample size for cohorts 1 and 2 was based on our previous behavioural studies using bowl digging tasks where we have performed a meta‐analysis and established a large effects size.^48^ There is also a relatively extensive literature for touchscreen based cognitive tests which suggests the similar large effect sizes and so used the same sample size. For cohorts 3 and 4, a larger number of animals was used due to stock available and to avoid animals which had been bred under the Animals in Scientific Procedures Act, UK, not delivering any scientific benefits. We also found some issues with some behavioural tests in this strain where the expected outcomes for the wild type controls was not achieved as described above supporting the potential benefit of using a larger sample size.

Rats were housed in groups of 2–4 in cages with ad libitum food and water with sawdust bedding with paper ribbon and enrichment in the form of hemp rope, chew block, plastic house, cardboard tube (cohort 1) and chew block and cardboard tube (cohorts 2–4). The animals were housed under reverse 12‐hour light (lights off at 08:15) and humidity‐ and temperature‐controlled conditions. Light food restriction was used for some tasks, with rat body weight maintained at over 90% their free‐feeding weight and their weight matched to a free‐feeding growth curve. Research was carried out under local institutional guidelines (approved by the University of Bristol and Cardiff University Animal Welfare and Ethical Review Boards) and in accordance with the UK Animals (Scientific procedures) Act 1986.

### 2.2 Behavioural tasks

The experimenter was blind to genotype throughout data acquisition and analyses. Task sequence across cohorts is summarised in Supplementary Figure S1. All behavioural testing was conducted during the light off phase, usually between 10:00 and 16:00 PM.

### 2.3 Bowl‐digging reversal learning task

All training and testing were run under red light conditions. Rats were habituated to handling and trained to dig using the standard protocol in the Robinson lab.^48^ Over 10 days, rats were handled daily for ~3 min in their home cages and tickled.^49^, ^50^ On days 11–12 days, rats were habituated to the arena (40 × 40 cm clear Perspex box) with 2 ceramic bowls (10 cm diameter) containing 45 mg sucrose enriched food reward pellets (Test Diet, Sandown Scientific, UK). On day 11, rats were placed into the arena with their cage mate for 10 min. On day 12, rats were placed into the arena alone for 10 min. On days 13–17, rats were placed into the arena alone and only one of the bowls was baited with reward pellets, with location counterbalanced over days. Rats had up to 30 s to retrieve the pellet. Retrieval failure incurred a trial restart, repeated until six consecutive trials were performed. Following retrieval success, the unrewarded bowl was removed and the rat removed from the arena after it disengaged from the bowl and the next trial set up. Throughout days 13–17, reward pellets were covered in increasing amounts of sawdust, encouraging digging. On day 17 rats were subjected to a discrimination test, where both bowls contained novel substrates (mouse bedding vs. shredded cloth) and where only one of the substrates was associated with reward. Successful discrimination occurred following the choice of the rewarded bowl for 6 consecutive times.

Food‐restricted rats were subjected to variations of the bowl‐digging reversal learning task (BRLT), a novel reversal learning task.^51^, ^52^, ^53^, ^54^, ^55^ Rats had the choice of digging in substrate‐containing bowls R or P, which were rewarded either in a deterministic (100/0) or a probabilistic (80/20) manner. Rats were deemed to have chosen their bowl when digging commenced. Scoring was performed online due to the nature of the task (the experimenter must determine when the choice has been made). Trial latencies were recorded online with a stopwatch. After choosing the rewarded “rich” bowl 6 consecutive times, the acquisition phase was deemed complete and the rule was reversed. The reversal phase was likewise deemed complete after 6 consecutive “rich” choices. Rats had up to 30 s to choose a bowl after being placed into the arena. The deterministic versions of the task were capped at 30 trials per phase, whereas the probabilistic version of the task were capped at 50 trials per phase. Every trial began with the rat placed into the centre of the arena. Once a bowl was chosen, the other bowl was quickly removed from the arena. Once disengaged from the chosen bowl the rat was removed from the arena and the next trial set up. Bowl position was counterbalanced from trial to trial in a pseudorandom order. The rewarded substrate was also counterbalanced across genotype. Both bowls had contained a crushed reward pellet to reduce the likelihood of the rats using olfactory cues. Data obtained in previous studies in the affective bias test also suggest animals do not use olfactory cues in bowl digging tasks undertaken using these methods (Supplementary Figure S2). If a rat started but failed to complete a phase, the rat was recorded as having completed the maximum number of trials to criteria to allow statistical comparisons between groups. Trials with omissions were recorded and restarted. There were few omissions across the different versions of the task and most animals had no omissions at all.

Further, this task was carried out in its substrate and spatial versions, where either a substrate or bowl position were rewarded. Both the substrate and spatial versions were done in both the deterministic and probabilistic manners, for a total of four different BRLTs. For convenience, the four versions of the BRLT will be referred to as substrate deterministic, substrate probabilistic, spatial deterministic and spatial probabilistic BRLT. In the substrate versions of the task, both bowls contained different substrates, such as coloured wood chip vs coconut fibre. In the spatial versions of the task, both bowls contained sawdust. Only one version of the task was run per day. Acquisition and reversal phases of each version were run on the same day.

### 2.4 Visual discrimination and reversal

Sound‐proof operant boxes (Med Associates Inc, USA) running K‐Limbic software (Conclusive Solutions Ltd., UK) were used for this task. The boxes contained a light, tone generator and reward magazine. Touchscreens were divided into 3 panels (9.2 × 13.4 cm). Training consisted of two stages. The first stage of training required the animal to touch the initiation square presented in the central panel to receive reward (45 mg Test Diet, Sandown Scientific, UK). The second stage of training required the animal to touch the initiation square presented in the central panel and to then touch either of the 2 side panels to receive reward. Both side panels were active simultaneously after initiation on the central panel during this training stage. All training sessions were capped at either 30 min or 120 trials, whichever occurred first. Performing more than 50 trials on 2 consecutive sessions resulted in the animal advancing onto the next stage of training. During visual discrimination testing, the initiation square was presented in the central panel, followed by the simultaneous presentation of ‘flower’ and ‘wheel’ stimuli on either side, side randomised across trials. Only one of the stimuli images was rewarded in a counterbalanced manner across subjects. Choosing the unrewarded stimulus resulted a 10 s timeout with lights on. Failure to choose a stimulus within 30 s of initiation was classed as an omission and resulted a 10 s timeout with lights on. A maximum of 30 min or 100 trials per session was allowed. Performance in excess of 80% accuracy on 2 consecutive sessions resulted in the reversal of the reward contingencies. No minimum trial number was set. Animals performed the task in darkness with the house light of the chamber only illuminated during the timeout.

### 2.5 Novel object and object location preference

Rats were habituated to an empty open‐top arena (length: 62 cm; width: 62 cm; height: 40 cm) for 10 min on 2 consecutive days. On the third habituation day, rats were again placed into the same arena for 10 min. An object was left for the rats to explore in the centre of the arena. This object was not reused in the sample and test phases of the task. Objects of varying colour, shape, and size were used. All objects were weighted to be impossible for the rats to displace. Replicas of each object were used across the different phases of the task to avoid odour contamination. Ethanol (70% v/v) was used to clean objects and the arena in between task phases and in between animals. The order in which the rats were tested in the novel object and object location tasks was counterbalanced across sex and genotype. Males and females were run in separate groups to avoid odour contamination. After completing their first task, each rat was subjected to an additional habituation day, identical to habituation days 1–2. Behaviour was recorded with an over‐head camera. Time spent exploring objects in sample and test phases was manually scored. Both novel object and object location tasks consisted of a sample and a test phase^56^ (Figure 3 A, B). In the sample phase of both tasks, two identical objects were placed 10 cm from the corners along a wall of the arena. The rat was placed in the arena facing the centre of the opposite wall. The rat was allowed 40 s of exploration of the objects, for a maximum of 4 min in the arena. Following a 5 min delay, the rat was replaced in the arena facing the centre of the opposite wall again and was allowed 3 min to explore. In the test phase of the novel object preference task, 1 object was a replica of the objects in the sample phase and one object was entirely new and different. In the test phase of the object location preference task, two replicas of the first items were used. One object was put into a location occupied by an object in the sample phase, whilst the second object was put into a novel location. Objects were always arranged in a diagonal configuration relative to each other in the test phase of the object location task. In the novel object preference task, the position of the novel and familiar objects in the test phase and the objects used as novel and familiar were counterbalanced across sex and genotype. In the object location preference task, the novel location was counterbalanced across sex and genotype. Exploratory behaviour was defined as the rat directing its nose to the object at a distance of less than 2 cm, with climbing on or touching the objects while looking elsewhere not included. Both the sample and test phase were carried out under low level white light (lux not recorded) to enable video recording for subsequent analysis.

### 2.6 Y‐maze spontaneous alternation

The task was carried out in a clear‐walled Perspex Y‐maze with a white wooden floor (Stem: 40 cm length, arms: 30 cm length, all compartment width: 12 cm; height: 23.5 cm). A clear Perspex roof was used to prevent escape. Male rats were run before female rats. Rats were habituated to the empty maze for 10 min per day over three consecutive days. Rats were allowed to explore the maze freely during this time, without experimenter intervention; this is the continuous version of the task. In the trial version of the task, a rat was placed in the start arm. Once the rat entered another arm, the door to that arm was closed. After a 30 s confinement in the chosen arm the rat was removed and placed in a holding cage in a neutral room for the 60 s ITI or back in the holding cage for the 24 h ITI before being placed back in the start arm. At each ITI rats submitted five trials each. Spontaneous alternations were calculated based on methods described in Miedel et al.^57^ For the continuous version of the task, alternation was defined as three different arm entries in three consecutive arm entries. Testing was carried out under white light conditions (110 lux) with and task performance was recorded for offline analysis.

### 2.7 Novelty suppressed feeding test

22 h after the removal of food, rats were individually exposed to a novel environment, a circular arena (height: 50 cm, diameter: 70 cm), containing standard lab chow in the centre. The mild stress of the novel environment delays chow consumption and it is this delay that is the main output of the test. 70% v/v ethanol solution was used to clean the arena between sessions. Feeding latency was recorded online with a stopwatch. Faecal pellets were counted online between tests. Testing was carried out under low level white light conditions (lux not recorded).

### 2.8 Modified progressive ratio

Sound‐proof operant boxes (Med Associates Inc, USA) running MED‐PC software were used for this task. The boxes were fitted with a response lever (3 cm above the floor) and animals were tested in the dark accept when the lever light was illuminated. Sucrose was accessible through drinking spouts that were automatically presented. Rats were trained to drink 16% sucrose with daily 15 min training sessions across 5 days. Rats had access to 3 epochs of 300 s sucrose access (day 1), five epochs of 2 × 60 s access separated with a 160 s period of no sucrose (day 2), and 5 epochs of 6 × 20 s access separated with a 55 s period of no sucrose (day 3). Rats then had 2 days of 5 epochs with 24 × 5 s access separated by a 25 s period of no sucrose. Subsequently, rats were trained to press the lever to receive sucrose rewards over 11 days, with one session a day. All sessions consisted of 7 × 5 min epochs (total session length 35 minutes). Firstly, a FR‐ 1 schedule was used where a single lever press delivered 5 s sucrose, followed by an ITI of 20 s where the lever was not available. The light above the lever turned on to indicate when it was baited and terminated in the ITI. It took five days of FR‐1 to learn to reliably press the lever (> 70 presses, ~ 10 per epoch). Rats were then trained on three days of a VR‐5 schedule and three days of a VR‐10 schedule to encourage continued pressing for reward. Rats learned to lever press for sucrose reward inconsistently, with a few subjects struggling at each training phase. For struggling rats, the reward ratio on the subsequent epoch was lowered to encourage lever pressing (i.e. from VR‐5 to VR‐2 or VR‐10 to VR‐7. Once rats reliably pressed at each variable ratio rats were tested in an ascending FR schedule. In this procedure the number of lever responses required for sucrose reward was increased every epoch on a quarter logarithmic scale (the number of lever presses required increased in the following manner: 1, 2, 3, 5, 10, 18, 32 as in Reference^58^). The FR schedule progressed regardless of epoch performance. During pretraining rats were maintained at 90% free‐feeding weight on a food restriction schedule. Rats were then tested with a 16% sucrose reward for four days while food restricted and for four days while not food restricted. Food restriction was counterbalanced for genotype, with half of the wild‐types and Dlg2+/− rats being restricted first and the other half non‐restricted first before this swapped in the repeated measures design.

### 2.9 Data analysis

Trial number was the primary output of BRLT and visual discrimination and reversal. Discrimination ratio was the primary output of novel object and object location tasks. Feeding latency was as the primary output of the novelty‐suppressed feeding task. The number of rewards obtained was the primary output of the modified progressive ratio task. The number of arm entries was the primary output of the spontaneous alternation task. Unpaired t‐tests were used for most tests to assess the effects of genotype. Where animals were not of uniform sex in a cohort, two‐way ANOVA was used to incorporate sex and genotype in the analysis. Repeated‐measures 3‐way ANOVA was used for the modified progressive ratio task. Where sex was not a significant factor, the effects of sex and the interactions of other factors with sex are reported in the supplementary material. *α* = 0.05 was applied for all tests null hypothesis significance testing (NHST) procedures. Findings were considered trends at P *<* 0.1 and are also discussed. NHST analyses were done on SPSS 21.0.0.0 and Graphpad Prism 7 softwares.

However, NHST procedures only provide a p value given the assumption of the null hypothesis, and thus they cannot provide evidence *for* the null hypothesis.^59^ In contrast, Bayesian tests calculate the probability of observing the current data relative to both the null and alternative hypotheses, thus affording an assessment of whether the data supports the null. Here, Bayesian statistics are used where a null result would be informative (most commonly, where there is potentially evidence for a no difference between genotypes). Bayes factors are probability ratio for the observed data under a model based on the null hypothesis compared with a model based on some specified alternative. When represented as BF01 Bayes factors vary between 0 and infinity, where 1 indicates that the data are equally consistent with either null or alternative, values greater than 1 indicate the data is more consistent with the null than the alternative hypothesis, while values less than 1 indicate the data is more consistent with the alternative than the null. The following conventions for interpretation suggested by Jeffreys et al.^60^ were followed: BF01 between 1 and 3 gives weak or anecdotal support to the null, BF01 between 3 and 10 represents some supporting evidence, while BF01 more than 10 indicates strong evidence for the null. Bayes factors were calculated for factorial ANOVAs in the way described by Rouder, Morey, Speckman, and Province^61^and Rouder, Morey, Verhagen, Swagman, and Wagenmakers^62^and were implemented using JASP 0.14.0 and the default prior scale for fixed and random effects and reported as the analysis of effects—this gives a BFexclusion (BFexcl) which is equivalent to BF01 when averaging across models including the factor or interaction of interest. Bayes factors for t‐tests were calculated as described by Rouder et al.^63^ and implemented using JASP 0.14.0 with both default (0.707) and wide (1) settings for the Cauchy prior distribution on effect size under the alternative hypothesis (in essence, this allows for an assessment of the degree to which the data are consistent with the null when assuming either a moderate or large alternative effect size). Figures were created using the Graphpad Prism 7, and Microsoft PowerPoint.

Win stay probability, lose shift probability, discrimination ratio, percentage alternation analysis learning data were calculated using the following formulae:

$$\mathrm{win}\;\text{stay probability}=\frac{\left(\text{rich rewarded stay count}+\text{lean rewarded stay count}\right)}{\left(\text{rich rewarded stay count}+\text{lean rewarded stay count}+\text{rich rewarded shift count}+\text{lean rewarded shift �count}\right)},�$$

$$\text{lose shift probability}=\frac{\left(\text{rich punished shift count}+\text{lean punished shift count}\right)}{\left(\text{rich punished shift count}+\text{lean punished shift count}+\text{rich punished stay count}+\text{lean puished stay �count}\right)},�$$

$$\text{Discrimination ratio}\left(D2\right)=\frac{\text{exploration of novel object or pair}-\text{exploration of familiar object or pair}}{\text{total exploration �time}},�$$

$$\text{percentage alteration}=\frac{\text{number of spontaneous alterations}}{\text{total number of}\;\mathrm{arm}\;\text{entries}-2}\times 100.$$

## 3 RESULTS

### 3.1 Bowl‐digging reversal learning task

**FIGURE 1:**
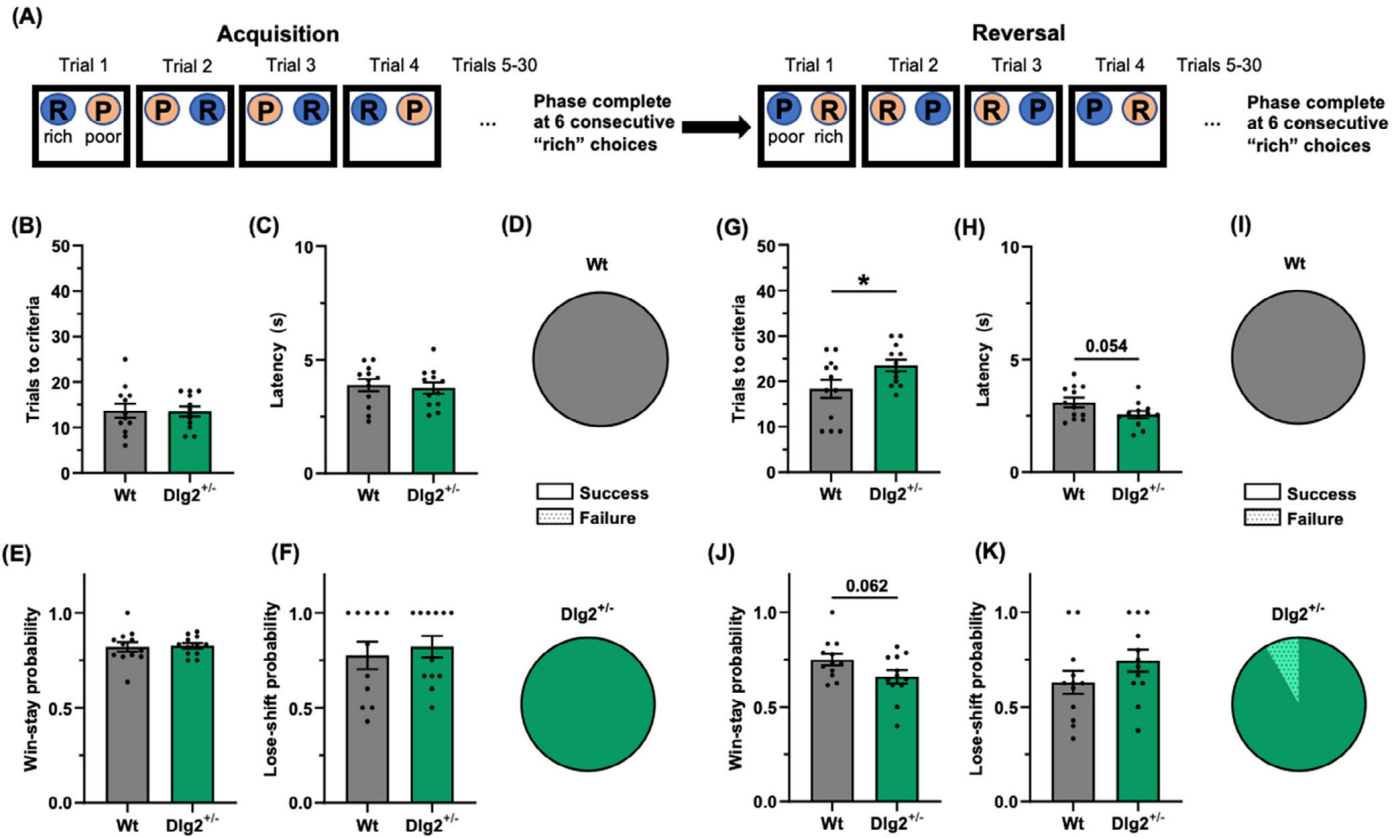
Dlg2+/− rats required more trials to reverse in the substrate deterministic, bowl‐digging reversal learning task. A) schematic overview of the substrate deterministic bowl‐digging reversal learning task, consisting of acquisition and reversal phases. Trials to criteria (B), latency (C), proportion of animals successfully completing the phase of the task (D), win‐stay (E) and lose‐shift (F) probabilities for the acquisition phase of the task. Trials to criteria (G), latency (H), proportion of animals successfully completing the phase of the task (I), win‐stay (J) and lose‐shift (K) probabilities for the reversal phase of the task. Summary values depicted as mean ± SEM. *P < 0.05 (unpaired t‐test).

Dlg2+/− and wild‐type controls of cohort 1 were tested in the substrate deterministic bowl‐digging reversal learning task (Figure 1 A). Animals of both genotypes were able to successfully complete the acquisition phase of the task, as indicated by comparable trials to criteria (Figure 1 B, D). There was no evidence for an effect of genotype in trials to criteria (t(22) = 0.09, *p* = 0.931, BF01(default) = 2.67, BF01(wide) = 3.47). Trial latency was similar across genotypes (t(22) = 0.37, *p* = 0.719, BF01(default) = 2.55, BF01(wide) = 3.29) (Figure 1 C). Likewise, there was no evidence for an effect of genotype on either the win‐stay (*t*(22) = 0.20, *p* = 0.847, BF01(default) = 2.64, BF01(wide) = 3.43) or the lose‐shift metrics (*t*(22) = 0.50, *p* = 0.621, BF01(default) = 2.41, BF01(wide) = 3.08) (Figure 1 E, F). Taken together, there is no evidence to suggest an alternation of the Dlg2+/− rat ability to discriminate between rewarded and unrewarded substrates in this task. All animals, with the exception of one Dlg2+/− rat, were able to successfully complete the reversal phase of the task (Figure 1 I). Whilst both Dlg2+/− and wild‐type rats required numerically more trials to achieve criteria in the reversal phase compared with the acquisition phase of the task, Dlg2+/− rats required more trials than their wildtype counterparts (t(22) = 2.16, p = 0.042), indicating a reversal learning impairment (Figure 1 G). There was a trend suggesting Dlg2+/− rats may have spent less time per trial compared to wild‐types (t(22) = 2.04, p = 0.054, BF01(default) = 0.64, BF01(wide) = 0.69) (Figure 1 H). There was also a trend suggesting Dlg2+/− rats may have exhibited greater win‐stay probability (t(22) = 1.96, p = 0.063, BF01(default) = 0.70, BF01(wide) = 0.78) (Figure 1 J). There was no evidence for an effect of genotype on lose‐shift probability (t(22) = 1.36, p = 0.187, BF01(default) = 1.38, BF01(wide) = 1.64) (Figure 1 K).

In an effort to challenge the animals, the substrate probabilistic version of this task was performed, where the substrate containing‐bowls were rewarded in a probabilistic manner (Supplementary Figure S3). Approximately three quarters of animals of both genotypes were able to successfully complete the acquisition phase of the task. Of those, fewer still were able to successfully complete the reversal phase of the task. There was no effect of genotype in trials to criteria during the acquisition (*t*(22) = 0.93, *p* = 0.361, BF01(default) = 1.95, BF01(wide) = 2.43) or the reversal phases of the task (*t*(15) = 1.125, *p* = 0.278, BF01(default) = 1.56, BF01(wide) = 1.86). Dlg2+/− rats had lower latency than their wild‐type counter parts in the acquisition phase of the task (*t*(22) = 2.295, p = 0.032). There was no effect of genotype on latency during the reversal phase of the task (t(15) = 1.569, p = 0.138, BF01(default) = 1.07, BF01(wide) = 1.21). There was a trend suggesting reduced win‐stay probability in the Dlg2+/− rats in the acquisition phase of the task (*t*(22) = 1.91, *p* = 0.069, BF01(default) = 0.75, BF01(wide) = 0.83). There was no effect of genotype on lose‐shift probability during the acquisition phase of the task (*t*(22) = 0.69, *p* = 0.498, BF01(default) = 2.25, BF01(wide) = 2.86). Notably, there was a numerical difference in win‐stay probability in the reversal phase of the substrate probabilistic version of the task (*t*(15) = 1.068, *p* = 0.302, BF01(default) = 1.62, BF01(wide) = 1.95) (Supplementary Figure S3), the direction of which matched the trend seen in the same parameter in the substrate deterministic version of the BRLT (Figure 1 J). There was a lose‐shift effect across genotype in the reversal phase of the substrate probabilistic version of the BRLT (t(22) = 4.15, p = 0.001) (Supplementary Figure S3), which also matched the numerical difference and direction seen in the substrate deterministic version of the task (Figure 1 K).

The performance of the rats in the spatial versions of the task was broadly similar to that in the substrate versions but there were some important differences. All rats of both genotypes successfully acquired the initial rule in the spatial deterministic version of the task. (Supplementary Figure S4). In contrast to the substrate deterministic version, in the spatial deterministic version of the task all animals of both genotypes were able to reverse following the rule change. There were no indications of differences across genotype in any of the measured factors, including trials to criteria (*t*(22) = 0.141, *p* = 0.889, BF01(default) = 2.66, and BF01(wide) = 3.45), latency (*t*(22) = 1.61, *p* = 0.122, BF01(default) = 1.07, and BF01(wide) = 1.24), win‐stay probability (t(22) = 0.69, *p* = 0.500, BF01(default) = 2.26, and BF01(wide) = 2.86), lose‐shift probability (*t*(17) = 0.54, *p* = 0.597, BF01(default) = 2.22, and BF01(wide) = 2.80) during the acquisition phase, and trials to criteria (t(22) = 0.80, p = 0.430, BF01(default) = 2.12, BF01(wide) = 2.67), latency (t(22) = 0.77, *p* = 0.452, BF01(default) = 2.16, and BF01(wide) = 2.73), win‐stay probability (t(22) = 0.531, *p* = 0.601, and BF01(default) = 2.42, and BF01(wide) = 3.09), lose‐shift probability (t(22) = 0.75, *p* = 0.461, BF01(default) = 2.18, and BF01(wide) = 2.76) during reversal the phase.

In the spatial probabilistic version of the task, most animals successfully completed the acquisition phase, with no indication of genotype‐specific effects on trials to criteria (*t*(22) = 0.24, *p* = 0.814, BF01(default) = 2.62, and BF01(wide) = 3.40), latency (*t*(22) = 0.69, *p* = 0.498, BF01(default) = 2.25, and BF01(wide) = 2.86), win‐stay probability (*t*(22) = 0.17, *p* = 0.871, BF01(default) = 2.65, and BF01(wide) = 3.44), lose‐shift probability (*t*(22) = 0.40, p = 0.693, BF01(default) = 2.53, and BF01(wide) = 3.26) (Supplementary Figure S5). Approximately, half of the animals of both genotypes were able to successfully complete the reversal phase. There was no indication of genotype‐specific effects in the reversal phase of this version of the task on trials to criteria (*t*(19) = 0.523, *p* = 0.607, BF01(default) = 2.32, BF01(wide) = 2.95), latency (*t*(19) = 0.55, *p* = 0.587, BF01(default) = 2.29, and BF01(wide) = 2.91), win‐stay probability (*t*(19) = 0.90, *p* = 0.378, BF01(default) = 1.92, and BF01(wide) = 2.37), lose‐shift probability (*t*(19) = 0.97, *p* = 0.346, BF01(default) = 1.84, and BF01(wide) = 2.26).

### 3.2 Visual discrimination and reversal

**FIGURE 2:**
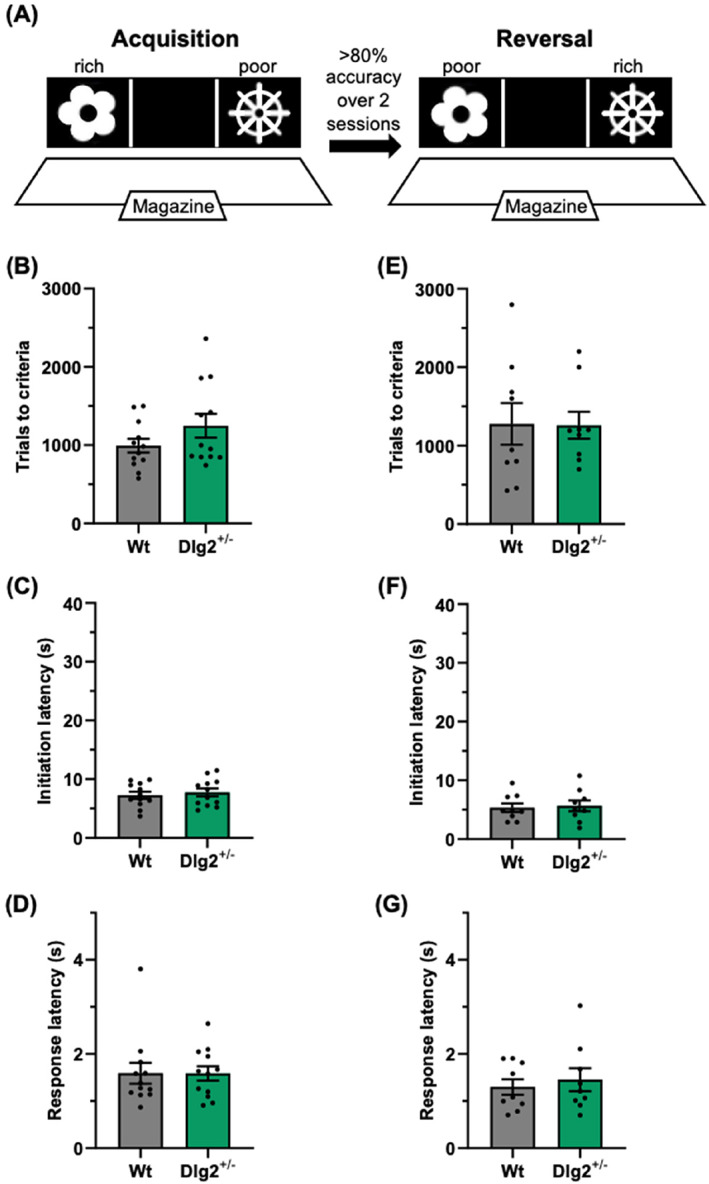
Unimpaired visual discrimination and reversal learning in Dlg2+/− rats. A) Schematic overview of the visual discrimination and reversal learning task, consisting of acquisition and reversal phases. Trials to criteria (B), initiation latency (C), and response latency (D) for the acquisition phase of the task. Trials to criteria (E), initiation latency (F), and response latency (G) for the reversal phase of the task. Summary values depicted as mean ± SEM.

The same cohort 1 of rats was subjected to the touchscreen visual discrimination and reversal task (Figure 2 A). There was no evidence for an effect of genotype on trials to criteria (*t*(22) = 1.45, *p* = 0.161, BF01(default) = 1.26, and BF01(wide) = 1.49), initiation latency (*t*(22) = 0.52, *p* = 0.612, BF01(default) = 2.43, and BF01(wide) = 3.12), or response latency (*t*(22) = 0.02, *p* = 0.987, BF01(default) = 2.68, and BF01(wide) = 3.48) in the acquisition phase of the task (Figure 2 B–D). Similarly, there was no evidence for an effect of genotype on trials to criteria (*t*(16) = 0.06, *p* = 0.957, BF01(default) = 2.43, and BF01(wide) = 3.11), initiation latency (*t*(16) = 0.27, *p* = 0.789, BF01(default) = 2.37, and BF01(wide) = 3.02), or response latency (*t*(16) = 0.52, *p* = 0.609, BF01(default) = 2.21, and BF01(wide) = 2.79) in the reversal phase of the task (Figure 2 E–G). Taken together, these results indicate healthy visual discrimination and reversal in the Dlg2+/− rats.

### 3.3 Novel object and object location preference

**FIGURE 3:**
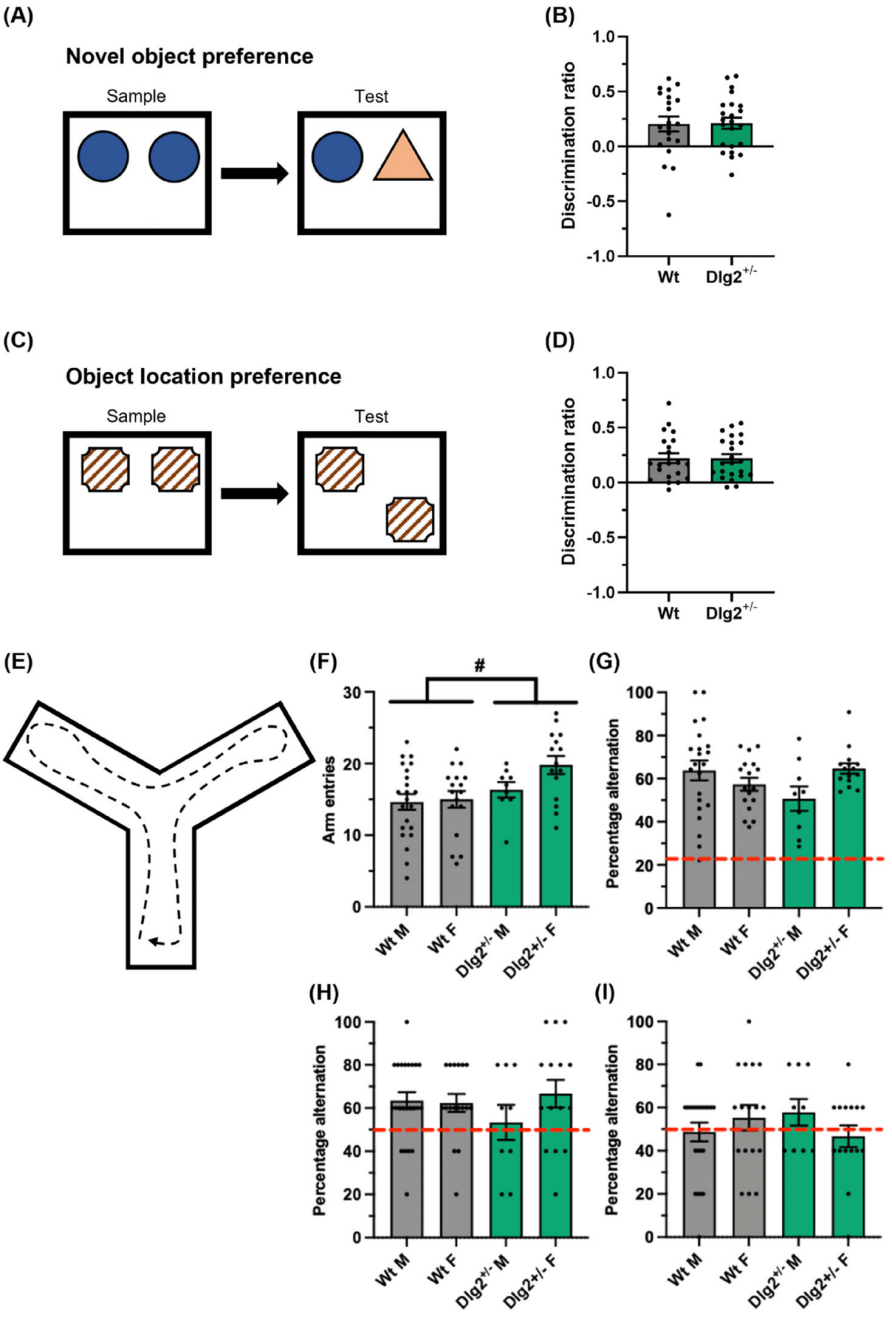
Unimpaired novel object preference, object location preference, and spontaneous alternation in Dlg2+/− rats. (A) Schematic overview of the novel object preference task. (B) Discrimination ratio in the novel object preference task. (C) Schematic overview of the object location preference task. (D) Discrimination ratio in the object location preference task. (E) Schematic overview of the spontaneous alternation task. Total arm entries (F), and percentage alternation (G) in the spontaneous alternation task (continuous version). Percentage alternation in the spontaneous alternation task (trial version) with a 1 min (H) and 24 h (I) intertrial interval. Chance percentage is indicated by the red dashed line: 2/9 in (G), 1/2 in (H) and (I). Summary values depicted as mean ± SEM. #*p* < 0.05 main effect of geneotype, 2‐way ANOVA.

Both wild‐type (*t*(20)) = 3.04, *p* = 0.009) and Dlg2+/− (t(22)) = 4.13, and *p* < 0.001) rats of cohort 3 were able to successfully discriminate in the novel object preference task (Figure 3 A, B). However, there was no indication of a genotype‐specific effect in this task (*F*_1,40_ = 0.063, *p* = 0.803, and BFexcl = 4.30). There was no effect of sex (*F*_1,40_ = 0.079, *p* = 0.780, and BFexcl = 4.25) and no sex‐genotype interaction (*F*_1,40_ = 1.904, *p* = 0.803, and BFexcl = 6.30).

Likewise, both wild‐type (*t*(20)) = 4.92, *p* = 0.001) and Dlg2+/− (*t*(22)) = 5.79, and *p* = 0.001) rats were able to successfully discriminate in the object location preference task (Figure 3 C, D). There was no genotype effect in this task either (*F*_1,40_ = 0.053, *p* = 0.819, and BFexcl = 4.28). There was no effect of sex (*F*_1,40_ = 1.636, *p* = 0.208, and BFexcl = 2.43) and no sex‐genotype interaction (*F*_1,40_ = 0.470, *p* = 0.497, and BFexcl = 7.04).

There were no differences observed for exploration in either the habituation phase or either the sample phase of the novel object or object in place tasks (Supplementary Figure S6).

### 3.4 Y‐maze spontaneous alternation

Cohort 4 rats of both genotypes also performed well in both the continuous and trial versions of the spontaneous alternation task (Figure 3 E). Dlg2+/− rats performed more arm entries than wild‐type rats did in the continuous version of the task (2‐way ANOVA: *F*_1,59_ = 6.562, and *p* = 0.013) (Figure 3 F). Percentage alternation was above the levels of chance for both wild‐type (*t*(38) = 13.34, *p* < 0.001) and Dlg2+/− (t(23) = 12.90, *p* < 0.001) rats in the continuous version of the task (Figure 3 G). However, there was no genotype effect in percentage alternation for the continuous version of the task (*F*_1,59_ = 0.443, *p* = 0.508, and BFexcl = 3.46) (Figure 3 G). There was no effect of sex (*F*_1,59_ = 0.781, *p* = 0.380, and BFexcl = 3.61) but there was a genotype‐sex interaction (*F*_1,59_ = 5.549, *p* = 0.0218, and BFexcl = 2.04) in percentage alternation.

Percentage alternation was above the levels of chance for both wild‐type (*t*(39) = 4.60, *p* < 0.001) and Dlg2+/− (t(23) = 2.29, *p* = 0.032) rats in the trial (1 min) version of the task (Figure 3 H). There was no genotype effect on percentage alternation in the trial (1 min) version of the task (*F*_1,60_ = 0.283, *p* = 0.597, and BFexcl = 4.74) (Figure 3 H). There was no evidence of a sex effect (*F*_1,60_ = 1.239, *p* = 0.270, and BFexcl = 4.07) or a genotype‐sex interaction (*F*_1,60_ = 1.738, *p* = 0.192, and BFexcl = 6.80) in the trial (1 min) version of the task.

Percentage alternation was not above the levels of chance for both wild‐type (*t*(39) = 0.43, *p* = 0.671) and Dlg2+/− (t(23) = 0.21, *p* = 0.836) rats in the trial (24 h) version of the task (Figure 3 I) suggesting no evidence for intact memory at this interval. Nevertheless, there was no genotype effect on percentage alternation in the trial (24 h) version of the task (*F*_1,60_ = 0.002, *p* = 0.968, Bfexcl = 4.77) (Figure 3 I). There was no evidence of a sex effect (*F*_1,60_ = 0.163, *p* = 0.688, and BFexcl = 4.91) or a genotype‐sex interaction (*F*_1,60_ = 2.509, *p* = 0.118, and BFexcl = 6.49) in the trial (24 h) version of the task.

### 3.5 Modified progressive ratio

**FIGURE 4:**
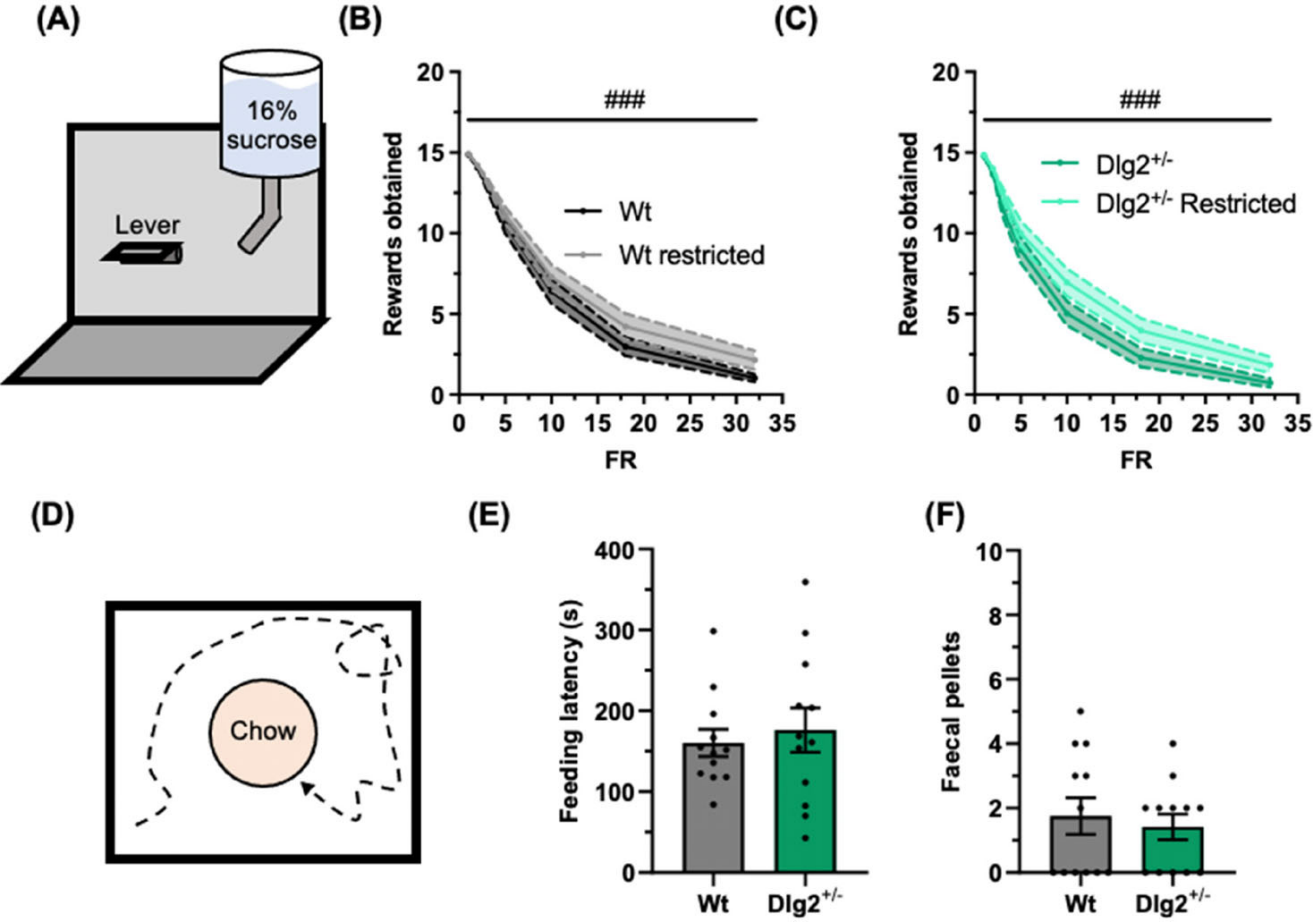
Unimpaired modified progressive ratio task and novelty suppressed feeding test in Dlg2+/− rats. (A) Schematic overview of the modified progressive ratio task. Rewards obtained across increasing fixed ratios with and without food restriction in wild‐type (B) and Dlg2+/− (C) rats. (D) Schematic overview of the novelty suppressed feeding test. Feeding latency (E) and number of faecal pellets (F) in the novelty suppressed feeding test. Summary values depicted as mean ± SEM. ###*P* < 0.001 (repeated‐measures 3‐way ANOVA).

Cohort 2 wild‐type and Dlg2+/− rats obtained progressively smaller numbers of rewards as the fixed ratio was increased in the modified progressive ratio task (*F*_6,276_ = 450.45, *p* < 0.001) (Figure 4 A–C). Food restriction successfully shifted this relationship upward (*F*_1,46_ = 29.44, *p* < 0.001). There was no indication of a genotype effect on obtained reward number (*F*_1,46_ = 1.61, *p* = 0.212, and BFexcl = 3.32) nor was there a genotype‐food restriction interaction (*F*_1,46_ = 1.70, *p* = 0.198, and BFexcl = 5.41).

### 3.6 Novelty suppressed feeding test

In the novelty supressed feeding test, there was no evidence for a genotype effect on feeding latency (*t*(22) = 0.49, *p* = 0.627, BF01(default) = 2.45, and BF01(wide) = 3.14) or faecal pellet number (*t*(22) = 0.48, *p* = 0.635, BF01(default) = 2.46, and BF01(wide) = 3.16) in cohort 1 rats (Figure 4 D–F).

## 4 DISCUSSION

We have recently demonstrated in the heterozygous Dlg2+/− rat model enhanced NMDA receptor currents, reduced input resistance, reduced dendritic arborisation, impaired dendritic integration, and associative plasticity,^33^ potentially suggesting impacts on hippocampal dependent learning and cognitive flexibility. Here, in addition to complementing the work done using full knockout models of Dlg2, we have expanded upon our recent molecular,^37^ physiological,^33^ and behavioural^37^ characterisation of the Dlg2+/− heterozygous rat model. Dlg2+/− rats exhibited a mild impairment in reversal learning in one of the naturalistic bowl‐digging reversal learning task. Their performance in the other forms of the bowl‐digging tasks, visual discrimination and reversal, novel object and object location tasks, spontaneous alternation, modified progressive ratio task, and novelty‐suppressed feeding test was unaffected. The naturalistic foraging tasks involve tests of acquisition and reversal learning which are performed within a single session and therefore involve different underlying behaviours to those performed in the touchscreen set‐up. They also involve a smaller number of trials to achieve learning criteria and are performed in sensory domains used by rodents for natural foraging behaviour. Although only one form of the bowl‐digging tasks revealed an impairment, these differences in task design may influence sensitivity to this mild impairment. The lack of effects in the other behavioural task carried out also suggest a relatively specific and subtle behavioural impairment in this genetic model of psychiatric disorder risk due to haploinsufficiency of Dlg2. The lack of effects in the novel object and spontaneous alternation tasks suggest there are no impairments in either recognition or working memory and the data from the NSF and progressive ratio tasks are consistent with a lack of effect on anxiety‐like behaviour or motivation. Whilst it should be noted that only this one aspect of cognition was altered amongst this battery of tests and may be a result of the number of tasks included, these data suggest that the Dlg2 mutation and associated hippocampal dysfunction impact on cognitive flexibility in specific circumstances.

In a reversal learning task with simple visual stimuli, Dlg2−/− mice have been reported to perform at control levels.^35^ In the same task but with complex stimuli, the performance of Dlg2−/− mice was impaired.^35^ The capacity of the Dlg2−/− animals to perform at wild‐type levels when learning a simple rule but to show impairments when the task is made more challenging, is reminiscent of the effects presented here. Although the Dlg2+/− rats here did not have an impairment in the acquisition phase of the substrate deterministic BRLT, they required more trials than the wild‐type rats did to complete the reversal phase, indicating a reversal impairment. We attempted to challenge the Dlg2+/− rats further by making the reward contingencies probabilistic, expecting to see a more pronounced difference across genotype. However, the task proved to be too difficult, as the wild‐type rats were largely unable to perform the task. Both spatial versions of the task appeared to be easier for animals of both genotypes, as greater numbers were able to complete the acquisition and reversal phases of both the deterministic and probabilistic versions of the task. These pieces of evidence could be interpreted as being indicative of intact hippocampal function in the Dlg2+/− model, despite altered hippocampal physiology previously reported in the same Dlg2+/− model^33^ and increased phencyclidine‐induced locomotion.^37^ In light of the consistent numerical difference and direction in win‐stay and lose‐shift probabilities in the substrate deterministic and probabilistic versions of the BRLT, it is possible there is a difference in the BRLT resolution strategy across genotype that spans both versions of the task. The possibility that the order in which the different versions of the BRLT were performed in played a role in task outputs should also be acknowledged, as the 4 versions of the BRLT were done in series.

Visual discrimination and reversal were intact in the Dlg2+/− rats here, similar to results from Dlg2−/− mice reported by Nithianantharajah et al.^35^ The reversal learning impairment seen in the Dlg2+/− rats in the substrate deterministic version of the BRLT is juxtaposed with the wild‐type level performance in the visual discrimination and reversal learning task. Although only one form of the bowl‐digging tasks revealed an impariment, one possible explanation is that naturalistic bowl‐digging associative learning versus touchscreen visual learning, have some fundamental but important differences. The sensory domain employed in the BRLT closely resembles that in natural foraging behaviour in rodents. It could be argued that tasks designed to incorporate or mimic naturalistic behaviour result in animals learning more quickly, with relatively few trials and/or sessions necessary to achieve criteria, as seen in a maze exploration task,^64^ virtual‐environment‐foraging task,^65^ and in an head‐fixed lick/no‐lick odour task.^66^ These tasks favour a more dynamic cognition and are less dependent on procedural learning. The findings of Nithianantharajah et al.,^35^ as well as those discussed here, together may be interpreted as being indicative of cognitive inflexibility in models of Dlg2 haploinsufficiency or knockout, a symptom seen in schizophrenia,^41^, ^44^, ^45^ autism spectrum disorder,^38^, ^39^ and attention deficit hyperactivity disorder,^40^, ^43^ for which genetic variations in Dlg2 are a risk factor for. Additionally, in the 5‐choice serial reaction time task Dlg2−/− mice were found to have lower accuracy and more premature responses.^35^ These findings align with the effects and trends of reduced latency in the Dlg2+/− rats in the substrate deterministic and substrate probabilistic versions of the BRLT presented here, with Dlg2+/− rats being slightly faster.

There was no indication of a genotype‐specific deficit in the Dlg2+/− rats in test of novel object and location preference, nor in spontaneous alternation. These findings could be interpreted as suggestive of grossly intact perirhinal cortex‐hippocampus function,^56^, ^67^, ^68^ despite altered NMDA receptor signalling and plasticity reported in this rat Dlg2+/− model.^33^, ^37^ Although food restriction did increase the motivation of rats to lever press for reward in the modified progressive ratio task here, there was no indication of a genotype‐specific effect, suggesting that motivation is preserved in the Dlg2+/− rats. Likewise, there was no difference across genotype in the novelty‐suppressed feeding test, which can be viewed as a measure of anxiety. As this test was performed by the same cohort of rats that were tested in the BRLT tasks and as no genotype‐specific deficit was reported in the Dlg2+/− rat model in open field and elevated plus maze,^37^ it can be interpreted that the impaired performance seen in the Dlg2+/− rats in the substrate deterministic BRLT is due to a cognitive impairment in reversal learning as opposed to a differential stress response. It remains difficult to draw conclusions about the mechanisms underlying impairments in reversal learning in the substrate deterministic BRLT. Based on our findings here and in Waldron et al. (2022), Dlg2+/− rats appear to have intact reward sensitivity, motivation, working memory, anxiety‐related behaviour, pre‐pulse inhibition, sociability, and largely intact learning and memory. If the numerical differences and directions of said differences in the win‐stay and lose‐shift probabilities in the substrate deterministic and probabilistic versions of the BRLT are tentatively accepted as trends, they could not readily be interpreted as arising from differences in reward sensitivity. It could be instead speculated that these putative differences stem from increased perseveration on the previously rewarded substrate. The reversal learning deficit reported here therefore resembles the cognitive deficits found in patients with schizophrenia and in patients with psychosis.^44^, ^45^, ^69^, ^70^ However, further studies using other behavioural tasks involving cognitive flexibility are needed before it can be determined how specific these impairments may be.

Our recent work indicated that increasing neuronal excitability by targeting either muscarinic M1 receptor or potassium channels^33^ can counteract the deleterious effects of the Dlg2 haploinsufficiency on input resistance, thereby rescuing dendritic integration and plasticity. It remains to be seen whether the systemic or local administration of the CNS‐penetrant and muscarinic M1 receptor specific agonist 77‐LH‐28‐1^71^ could ameliorate the reversal deficit seen here in the substrate deterministic BRLT. The developmental time course of the deficits in this model also remains to be investigated both at the levels of physiology and behaviour. It is also possible that if challenged further, with the addition of an environmental factor such as inflammation or maternal separation, Dlg2+/− models could become impaired to a greater extent than their wild‐type counterparts, potentially in a non‐linear gene x environment interaction.

## 5 CONCLUSION

Recent studies have reported altered neuronal function and behaviour in rodent models of Dlg2 knockout and haploinsufficiency. Here, we expand upon this work by specifically focusing on the rat Dlg2+/− heterozygous model. We build upon the behavioural characterisation of this model by extending the array of tasks, with a particular focus on reversal learning. Complementing our recent studies on Dlg2+/− rat behaviour^37^ and brain circuit function,^33^ we report a specific mild impairment in reversal learning one form of a naturalistic bowl‐digging reversal learning task in the Dlg2+/− rat model. There was no evidence for deficits in other bowl‐digging tasks involving spatial or probabilistic learning, touchscreen visual discrimination and reversal, novel object preference, novel location preference, spontaneous alternation, modified progressive ratio, and novelty‐suppressed feeding test in this Dlg2+/− rat model. Our findings suggest that the reversal learning impairment is unlikely to be confounded by altered stress responsiveness, motivation, working memory or memory of object identity and location. Cognitive impairment in the form of deficits in reversal learning seen in the Dlg2+/− rat model here may resemble some of the cognitive impairments observed in patients with schizophrenia and in patients with psychosis.

## FUNDING INFORMATION

Funding was provided by grants from Medical Research Council (UK) (GW4 BIOMED PhD studentship to SG), Biotechnology and Biological Science Research Council (UK) (JRM) and Wellcome Trust (UK) (JRM, PhD studentship to SW).

## CONFLICT OF INTEREST STATEMENT

Emma S. J. Robinson has received research funding from Boehringer Ingelheim, COMPASS Pathways, Eli Lilly, IRLab Therapeutics, Pfizer, Small Pharma Ltd. and MSD, and Dominic M. Dwyer has received research funding from Eli Lilly, but these companies were not associated with the data presented in this manuscript.

## ETHICS STATEMENT

All studies were approved by the local Animal Welfare and Ethical Review Bodies at the University of Bristol or University of Cardiff and carried out under Home Office Licences.

## Supporting information


**Data S1:** Supporting Information

## Data Availability

The full data set can be accessed via the Robinson lab open science framework https://osf.io/bhs82/
