# Supplementary material for: A mild impairment in reversal learning in a bowl‐digging substrate deterministic task but not other cognitive tests in the Dlg2+/− rat model of genetic risk for psychiatric disorder
Source: Genes Brain Behav. 2023 Sep 13;22(6):e12865. doi: 10.1111/gbb.12865 (PMC10733576; doi:10.1111/gbb.12865)
Supplement: Supplementary file 1 — Data S1: Supporting Information [file GBB-22-e12865-s001.docx]

**Supplementary information**

**A mild impairment in reversal learning in a bowl-digging substrate deterministic task but not other cognitive tests in the Dlg2+/- rat model of genetic risk for psychiatric disorder**

**Authors and affiliations**

Simonas Griesius 1, †, Sophie Waldron 2, 5, †, Katie A. Kamenish 1, Nick Cherbanich 1, Lawrence S. Wilkinson 2, 3, 5, Kerrie L. Thomas 2, 4, Jeremy Hall 2, 3, 4, Jack R. Mellor 1, Dominic M. Dwyer 2, 5, Emma S. J. Robinson 1,*

1 Centre for Synaptic Plasticity, School of Physiology, Pharmacology and Neuroscience,

University of Bristol, University Walk, Bristol BS8 1TD, UK

2 Neuroscience and Mental Health Research Institute, 3 MRC Centre for Neuropsychiatric

Genetics and Genomics, Schools of 4 Medicine and 5 Psychology, Cardiff CF24 4HQ, UK

† These authors contributed equally: Simonas Griesius, Sophie Waldron

* Corresponding author: Emma.S.J.Robinson@bristol.ac.uk

Contents

Supplementary Figure S 1

Supplementary Figure S 2

Supplementary Figure S 3

Supplementary Figure S 4

Supplementary Figure S 5

Supplementary Figure S 6


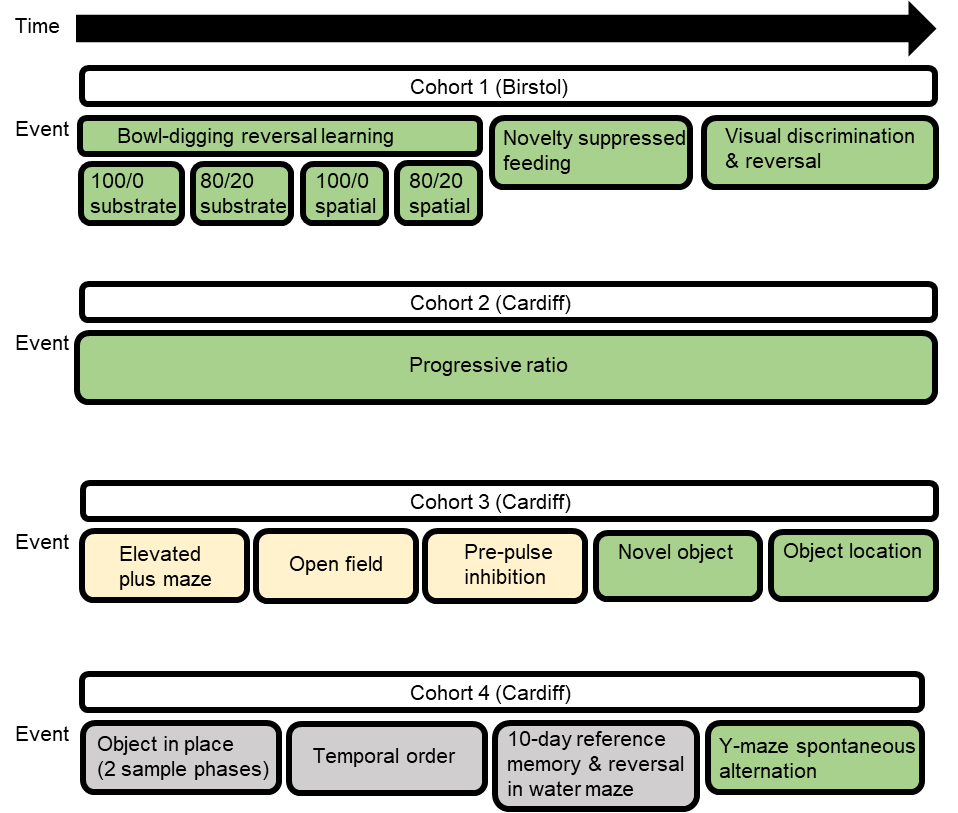


Supplementary Figure S 1 Schematic overview of 4 rat cohorts across the two research institutions, detailing which cohorts did which tasks and the sequence of tasks. Cohort numbers do not indicate the order in which cohorts were generated. Tasks highlighted in green are presented and discussed in the present study. Tasks highlights in yellow, elevated plus maze, open field, and pre-pulse inhibition tasks, are published in Waldron et al. 2022^35^ and are not discussed in this article but are presented here for clarity of experimental sequence. Data were only included for full analysis where the wild-type controls performed to the expected level. Data from object in place, temporal order, and 10-day reference memory & reversal in water maze tasks, highlighted in gray, were not included. In the object in place and temporal order tasks the wild type animals failed to show a positive discrimination ratio towards the novel or recent object. In the water maze tasks, the wild-type animals did not show significant learning or evidence of a memory for the platform.

Supplementary Figure S 2 data from two weeks of an affective bias test experiment (Hinchcliffe et al., 2019) which is run using a similar protocol to the studies described here but includes a test session with random reinforcement where animal receive a reward from either of the presented substrates at a rate of 1 in 3 over 30 trials. Chance performance is 10 rewards which is indicated in the figure with a dashed red line. Animals overall perform at chance level suggesting they are not using olfactory cues to solve the task.


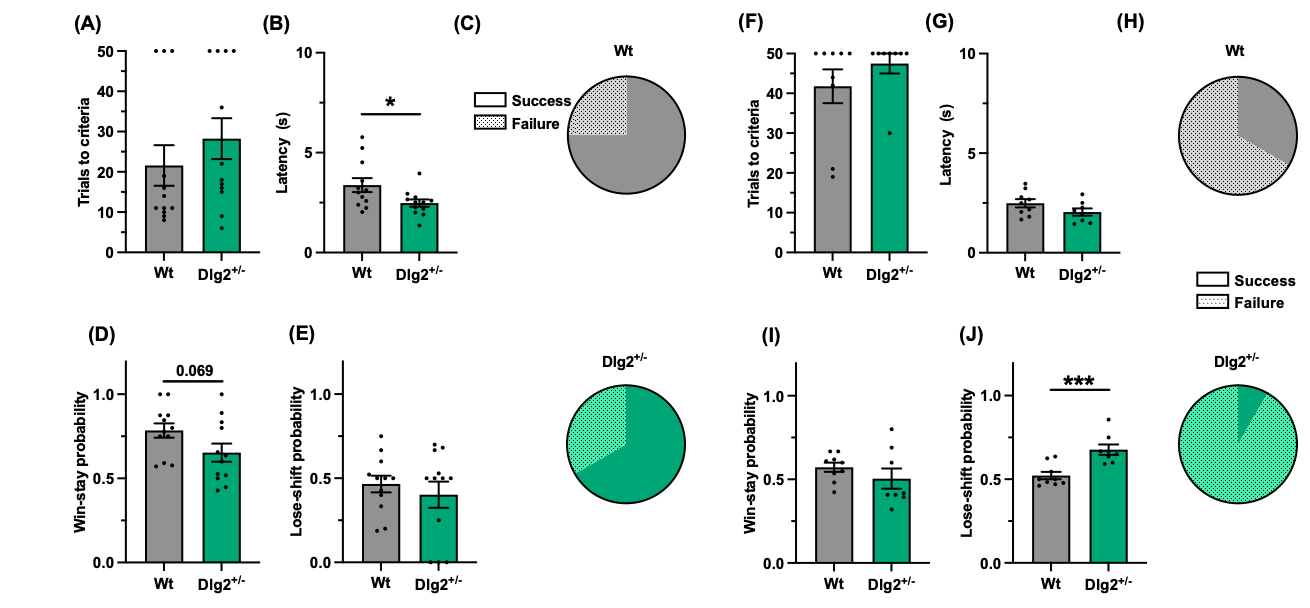


Supplementary Figure S 3 substrate probabilistic bowl-digging reversal learning task. Trials to criteria (A), latency (B), proportion of animals successfully completing the phase of the task (C), win-stay (D) and lose-shift I probabilities for the acquisition phase of the task. Trials to criteria (F), latency (G), proportion of animals successfully completing the phase of the task (H), win-stay (I) and lose-shift (J) probabilities for the reversal phase of the task. Summary values depicted as mean ± SEM. **P* < 0.05, ***P* < 0.01, ****P* < 0.001 (unpaired t-test).


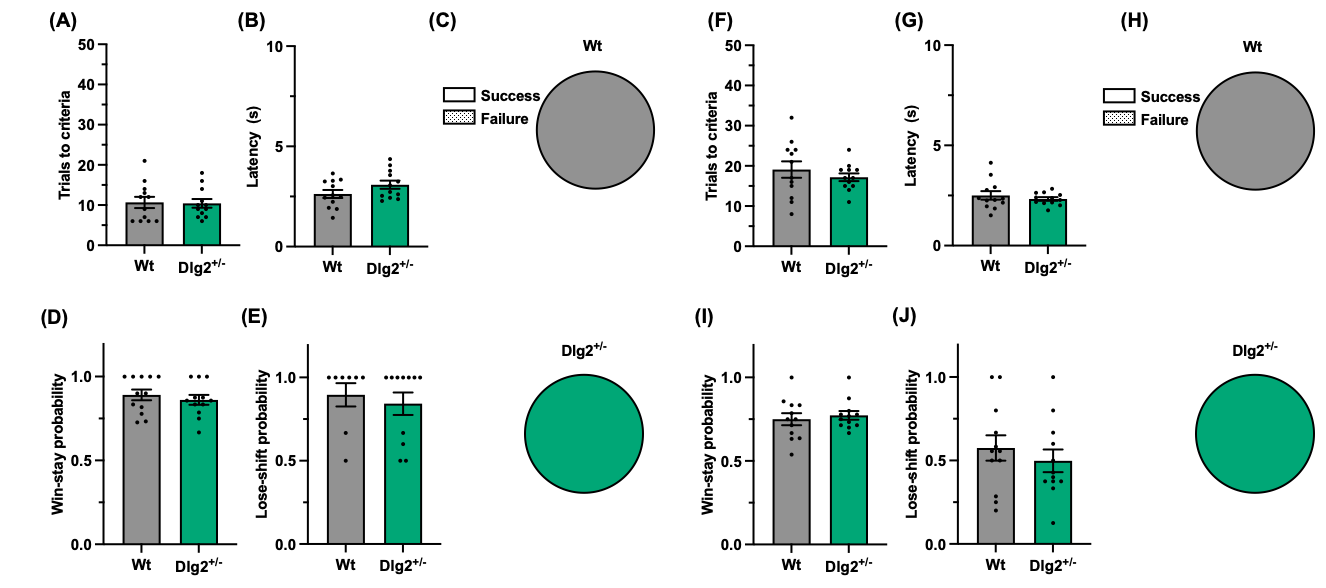
Supplementary Figure S 4 spatial deterministic bowl-digging reversal learning task. Trials to criteria (A), latency (B), proportion of animals successfully completing the phase of the task (C), win-stay (D) and lose-shift (E) probabilities for the acquisition phase of the task. Trials to criteria (F), latency (G), proportion of animals successfully completing the phase of the task (H), win-stay (I) and lose-shift (J) probabilities for the reversal phase of the task. Summary values depicted as mean ± SEM. **P* < 0.05, ***P* < 0.01, ****P* < 0.001 (unpaired t-test).


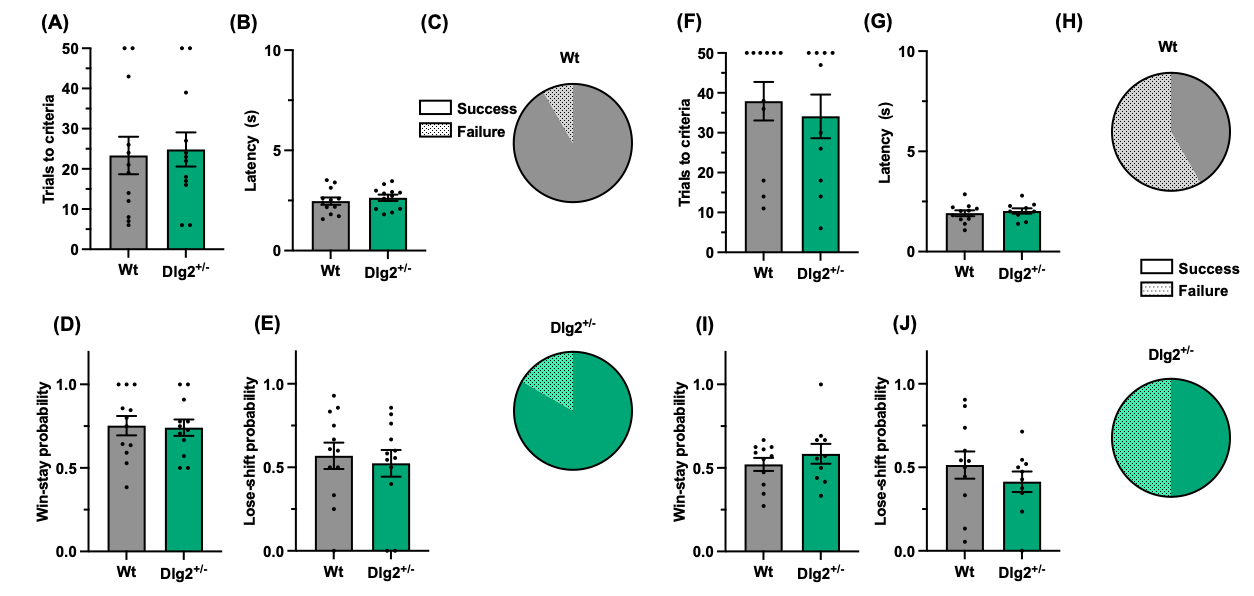
Supplementary Figure S 5 spatial probabilistic bowl-digging reversal learning task. Trials to criteria (A), latency (B), proportion of animals successfully completing the phase of the task (C), win-stay (D) and lose-shift (E) probabilities for the acquisition phase of the task. Trials to criteria (F), latency (G), proportion of animals successfully completing the phase of the task (H), win-stay (I) and lose-shift (J) probabilities for the reversal phase of the task. Summary values depicted as mean ± SEM. **P* < 0.05, ***P* < 0.01, ****P* < 0.001 (unpaired t-test).


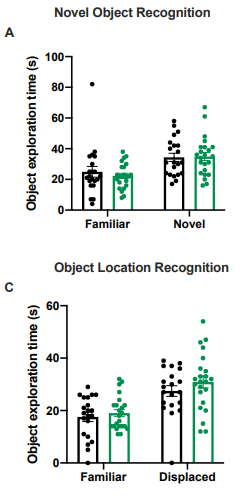


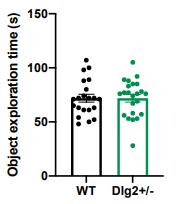


Supplementary figure S 6: There was no effect of genotype on object exploration time during the habituation phase (leftmost panel). There were no effects of genotype on object exploration times in the sample phases of the novel object and object location preference tasks.
